# Supplementary material for: Complexome profiling of the Chlamydomonas psb28 mutant reveals TEF5 as an early PSII assembly factor
Source: Plant Cell. 2025 Mar 17;37(6):koaf055. doi: 10.1093/plcell/koaf055 (PMC12164590; doi:10.1093/plcell/koaf055)
Supplement: koaf055_Supplementary_Data [file koaf055_supplementary_data.zip › psb28_interactive.zip!/plots/intensity/qualityPlots/peptides/NUOS5_Cre12.g511200.t1.2.html]

### Cre12.g511200.t1.2

Trivial name: NUOS5  
SeqCoverage [median]: 32.10  
Mass: 9.55 kDa  
  
 

| Sequence | MissedCleavage | Mass [Da] | wtIntensity | muIntensity | Score |
| --- | --- | --- | --- | --- | --- |
| AGMGLSTGTAR | 0 | 1020.50 | 401.21 | 977.59 | 59.24 |
| RAQLAGGSKPAGGGH | 2 | 1362.71 | 415.88 | 946.19 | 93.65 |
|  |  |  | 817.1 | 1923.8 |  |
